# Macrophage migration inhibitory factor in cerebrospinal fluid from patients with central nervous system infection

**DOI:** 10.1186/cc7933

**Published:** 2009-06-26

**Authors:** Christian Østergaard, Thomas Benfield

**Affiliations:** 1 Department of Clinical Microbiology, Copenhagen University Hospital Herlev, Herlev Ringvej, DK-2730 Herlev, Denmark; 2 Department of Infectious Diseases and Clinical Research Centre, Hvidovre University Hospital, Kettegårds Alle, 2650 Hvidovre, Denmark; 3 Faculty of Health Sciences, University of Copenhagen, Blegdamsvej, 2200 Copenhagen N, Denmark

## Abstract

**Introduction:**

Macrophage migration inhibitory factor (MIF) plays an essential pathophysiological role in septic shock, but its role in central nervous system infection (CNS) remains to be defined.

**Methods:**

We investigated cerebrospinal fluid (CSF) levels of MIF in 171 patients who were clinically suspected of having meningitis on admission. Of these, 31 were found to have purulent meningitis of known aetiology, 20 purulent meningitis of unknown aetiology, 59 lymphocytic meningitis and 11 encephalitis, whereas 50 were suspected of having but had no evidence of CNS infection.

**Results:**

CSF MIF levels were significantly higher in patients with purulent meningitis of known aetiology (median [interquartile range]: 8,639 [3,344 to 20,600] ng/l) than in patients with purulent meningitis of unknown aetiology (2,209 [1,516 to 6,550] ng/l; Mann-Whitney test, *P *= 0.003), patients with lymphocytic meningitis (1,912 [1,302 to 4,105] ng/l; *P *< 0.001) and patients suspected of having but without evidence of CNS infection (1,472 [672 to 3,447] ng/l; *P *< 0.001). Also, patients with encephalitis (6,937 [3,961 to 8,353] ng/l) had higher CSF MIF than did patients without CNS infection (*P *< 0.01). Among patients with purulent meningitis, CSF MIF levels were significantly higher in patients infected with pneumococci than in those with meningococcal infection (11,569 [8,615 to 21,935] ng/l versus 5,006 [1,717 to 10,905] ng/l; *P *= 0.02), in patients who required versus those not requiring assisted ventilation (10,493 [5,961 to 22,725] ng/l versus 3,240 [1,563 to 9,302] ng/l; *P *= 0.003), and in patients with versus those without impaired consciousness (8,614 [3,344 to 20,935] ng/l versus 2,625 [1,561 to 7,530] ng/l;* P *= 0.02). CSF MIF levels correlated significantly with meningeal inflammation (*P *< 0.05) but not with systemic inflammatory response (*P *> 0.05) in patients with purulent meningitis of known aetiology, those with lymphocytic meningitis and those with encephalitis.

**Conclusions:**

MIF was significantly increased in the CSF of patients with purulent meningitis and encephalitis, and was to some degree associated with severity of the infection. Our findings indicate that MIF may play an important role in CNS infection.

## Introduction

Macrophage migration inhibitory factor (MIF) is a cytokine that participates in both innate and adaptive immune responses. It is released from a wide range of cells, including macrophages and T lymphocytes, and when released it counter-regulates the inhibitory effect of corticosteroids on the release of pro-inflammatory cytokines from lipopolysaccharide-stimulated monocytes. This suggests a role of MIF in both the initiation of and sustaining the inflammatory cascade (for review see [1,2]). The role played MIF has been described extensively in sepsis, in which serum levels of MIF were found to be increased in septic patients and correlated with both disease severity and mortality [3-5]. Moreover, injection of MIF increased mortality in experimental sepsis, whereas inhibition of MIF decreased mortality [3,6], emphasizing the critical involvement of MIF in the pathophysiology of septic shock.

Bacterial meningitis is characterized by an overwhelming inflammatory cascade, primarily localized to the subarachnoidal space, which continues to evolve after eradication of the bacterial pathogen by antibiotic therapy [7]. Anti-inflammatory therapy with corticosteroids is beneficial in bacterial meningitis [8], suggesting that MIF may also influence the course of central nervous system (CNS) infections. Cerebrospinal fluid (CSF) levels of various inflammatory mediators have to some degree been useful as diagnostic and prognostic markers in meningitis [9-11]. Screening pooled CSF samples with microarray analysis for several mediators of the host immune response [12] revealed that MIF was upregulated in patients with purulent meningitis as compared with uninfected control individuals, indicating that MIF could be a potential new candidate for future meningitis studies. Moreover, MIF were elevated in CSF from patients with encephalitis due to West Nile virus [13] or with various neurological diseases such as multiple sclerosis [14] and Alzheimer's disease [15]. Furthermore, inhibition of MIF protected mice from brain disease due to West Nile virus [13] or autoimmune encephalomyelitis [16,17], further suggesting that MIF plays a pathophysiological role in CNS infection.

The aim of the present study was to investigate CSF MIF levels in 171 patients clinically suspected of having meningitis on admission and to study the use of CSF MIF in the differential diagnosis of meningitis and as a prognostic factor.

## Materials and methods

### Study design

Since 1988 CSF samples have been collected prospectively from patients undergoing lumbar puncture at the Department of Infectious Diseases at Hvidovre University Hospital. All lumbar punctures were performed as routine diagnostic procedures and were done in accordance with the hospital's ethical standards. Immediately after lumbar puncture, all CSF samples were transported directly to the Department of Clinical Biochemistry, where they were centrifuged, and the supernatants were collected and stored at -20°C within 15 to 30 minutes. The following day, CSF samples were registered and transferred for storage at -80°C. Because of the non-interventional nature of the study, blood samples were not routinely collected together with the CSF sampling. However, in six patients a blood sample was available that was obtained on admission and at the same time as the CSF sample. All protocols were approved by the local scientific ethics committee and the Danish Data Protection Agency (1995–1200/95–517); because the study required no intervention in addition to routine care, informed consent from patients was not required.

Patients' demographic and clinical characteristics, as well as biochemical and microbiological data, were obtained retrospectively from medicals records. All patients who were clinically suspected of having meningitis, in whom CSF samples was available that had been obtained at admission from a diagnostic lumbar puncture conducted during the period from 1988 to 2002, were included, except patients infected with HIV or *Mycobacterium tuberculosis *infection.

A total of 205 patients were identified. However, 34 patients (13 patients with purulent meningitis, 10 with lymphocytic meningitis, two with encephalitis and nine without CNS infection) were excluded, because MIF could not be measured for lack of CSF. Thus, the study comprised 171 patients. Based on clinical, microbiological and biochemical characterization, patients could be divided into five diagnostic groups.

The first group included those with purulent meningitis of known bacterial aetiology (n = 31). Twenty-five patients had positive CSF Gram stain or CSF culture (CSF white blood cell [WBC] count, median [range]: 3,857 [16 to 21,745] cells/μl) and six had neutrophil pleocytosis (CSF WBC count: 2,165 [16 to 18,485] cells/μl) and positive blood culture or a significant increase in antibody titres against *Neisseria meningitidis*. Fifteen cases were due to *N. meningitidis*, 11 to *Streptococcus pneumoniae*, two to *Haemophilus influenzae*, one to *Klebsiella pneumoniae*, one to *Staphylococcus aureus *and one to *Listeria monocytogenes*.

Patients in the second group, purulent meningitis of unknown bacterial aetiology (n = 20), had negative CSF and blood cultures, neutrophil pleocytosis (CSF WBC count: 359 [63 to 6,567] cells/μl, with > 80% neutrophils, except for one patient with 1,210 cells/μl and 45% neutrophils), who were treated for bacterial meningitis in accordance with local recommendations.

Five of the 20 patients (20%) with negative cultures and one out of the 31 patients (3%) with known bacterial aetiology had CSF WBC count under 1,000 cells/μl, CSF/blood glucose ratio above 0.3, CSF protein levels under 1 g/l and blood WBC count under 12 × 10^9 ^cells/l. Antibiotic therapy was initiated before the lumbar puncture in 23% (7/31) of patients with purulent meningitis of known bacterial aetiology and in 15% (3/20) of those with an unknown bacterial aetiology. The initial or empirical antibiotic therapy for purulent meningitis was intravenous ceftriaxone and ampicillin. If bacteria were demonstrated in the CSF and/or blood, then antibiotic therapy was changed in based on the susceptibility profile of the pathogen. The duration of antibiotic therapy was usually 7 to 10 days.

The third group included patients with lymphocytic meningitis (pleocytosis with a predominance of mononuclear cells; n = 59). All of these cases were due to acute aseptic meningitis. A known viral aetiology was established in 15 cases (enterovirus in 14 and herpes simplex virus-2 in one). The majority of patients recovered fully without antibiotic treatment, except for 14 patients who received one dose of antibiotics immediately after the lumbar puncture was performed and before the results of the CSF analysis were known (usually < 30 minutes after lumbar puncture). Two patients also received one dose of aciclovir.

The fourth group included patients with encephalitis (altered consciousness, abnormal electroencephalogram and/or computed tomography/magnetic resonance scan; n = 11). A viral aetiology was established in two cases (herpes simplex virus and varicella zoster virus [VZV]). All patients were treated with aciclovir (median 10 days, range 6 to 14 days).

The fifth diagnostic group included patients suspected of having meningitis but without evidence of CNS infection (no CSF pleocytosis; n = 50). Four patients had septic shock (in three this was due to *N. meningitidis *and in one it was due to *Escherichia coli*), seven had pneumonia, three had acute tonsillitis, one had acute otitis media, two had urinary tract infection, one had hepatitis due to *cytomegalovirus*, 25 patients had fever of unknown origin and seven had cephalgia.

### Cerebrospinal fluid analysis

CSF samples were analyzed by routine laboratory methods to determine glucose and protein levels, total leucocyte count and differential count.

### Measurement of MIF

MIF levels wee measured using a commercial available ELISA (R&D Systems, Inc. Minneapolis, MN, USA), in accordance with the manufacturer's instructions. In brief, MaxiSorp plates (Nunc, Roskilde, Denmark) were coated and incubated overnight at 4°C with a murine monoclonal anti-MIF antibody (R&D Systems), diluted in phosphate-buffered saline (PBS; Statens Serum Institut, Copenhagen, Denmark) to a final concentration of 2 μg/ml. After the plates were washed three times with wash buffer (PBS with Triton X-100), the unbound sites were blocked by adding 300 μl blocking buffer (PBS with 1% bovine serum albumin and 5% sucrose) for 90 minutes at room temperature. After another three washes, standards (recombinant human MIF [R&D Systems] in triplicates) or CSF and blood samples (at least in duplicate) diluted in dilution buffer (Tris-buffered saline with 0.1% bovine serum albumin and 0.05% Tween; Statens Serum Institut) were added and incubated for 2 hours at room temperature. After another three washes, MIF was detected by adding a biotinylated goat anti-human MIF antibody (R&D Systems) diluted in dilution buffer to a final concentration of 0.2 μg/ml and incubating for 2 hours at room temperature. After washing five times, streptavidin horseradish peroxidase (R&D Systems) diluted 1:1,000 in dilution buffer was added and incubated for 20 minutes at room temperature with subsequent washing five times and adding substrate solution, tetramethylbenzidine (KEM EN TEC Diagnostics, Taastrup, Denmark), for 30 minutes. The reaction was stopped with 1.2 mol/l H_2_SO_4_; Statens Serum Institut), and optical density at 450 nm was read on an ELISA reader. Lower limit of detection was 20 pg/ml.

### Statistical analysis

All data are expressed as medians and interquartile ranges. For continuous data, comparisons between two groups were performed using the Mann-Whitney test, whereas the Kruskal-Wallis test was used for comparisons between more than two groups. Fisher's exact test was used for comparisons between categorical data. When appropriate, correction with a Bonferroni's coefficient of 10 was used to compensate for multiple comparisons between the five diagnostic groups. For correlation analysis, the nonparametric Spearman's test was used. *P *< 0.05 were considered statistically significant.

## Results

### Clinical and demographic data for 171 patients in whom cerebrospinal fluid samples were taken on admission

Clinical and demographic data for the 171 patients with available CSF samples on admission are shown in Table 1. Twelve patients died during hospitalization (four with *S. pneumoniae *meningitis, one with *N. meningitidis *meningitis, one with *S. aureus *meningitis, one with *K. pneumoniae *meningitis, one with purulent meningitis of unknown aetiology, two with encephalitis of unknown aetiology, and two without meningitis [meningococcaemia and pneumonia]).

**Table 1:** Clinical and biochemical characteristics of 171 patients suspected of having meningitis on admission

	Purulent meningitis of known aetiology (n = 31)	Purulent meningitis of unknown aetiology (n = 20)	Lymphocytic meningitis (n = 59)	Encephalitis (n = 11)	No CNS infection (n = 50)
Female sex	45% (14/31)	50% (10/20)	41% (24/59)	45% (5/11)	48% (24/50)
Age (in years)	20 (10–53) (31/31)	23 (7–30) (20/20)	25 (17–31)^‡ ^(59/59)	55 (46–64) (11/11)	28 (10–44) (50/50)
Underlying illness*	23%^† ^(7/31)	10% (2/20)	2% (1/59)	0% (0/11)	4% (2/50)
Body temperature (°C)	38.8 (38.4–39.6)^† ^(27/31)	38.3 (38.0–39.2) (18/20)	38.1 (37.7–38.7) (49/59)	37.8 (37.1–38.9) (9/11)	39 (37.5–39.6) (37/50)
Mean arterial pressure (mmHg)	93 (87–117) (22/31)	90 (79–106) (12/20)	90 (82–100) (42/59)	102 (86–115) (10/11)	93 (77–103) (30/50)
Heart rate (beats/minute)	113 (98–129)^†‡ ^(20/31)	90 (80–108) (16/20)	88 (73–100) (44/59)	80 (70–101) (10/11)	100 (85–120) (30/50)
Septic shock	16%^† ^(5/31)	0% (0/20)	0% (0/59)	0% (0/11)	8% (4/50)
Back rigidity	80%* (24/30)	95%^‡§ ^(18/19)	88%^‡§ ^(51/58)	40% (4/10)	57% (26/46)
Decreased consciousness	66%*^†§ ^(19/29)	22%^†‡ ^(4/18)	0%^‡ ^(0/57)	91%^§ ^(10/11)	13% (6/46)
Assisted ventilation	45%*^†§ ^(14/31)	0% (0/20)	0% (0/59)	20% (2/10)	10% (5/50)
Steroid therapy	35%*^†§ ^(11/31)	0% (0/20)	0% (0/59)	10% (1/10)	0% (0/50)
Fatal outcome	23%^† ^(7/31)	5% (1/20)	0% (0/59)	18% (2/11)	4% (2/50)
Days in hospital (survivors)	11 (9–15)^†§ ^(24/24)	9 (8–12)^†§ ^(19/19)	4 (2–5)^‡ ^(59/59)	12 (11–30)^§ ^(9/9)	4 (2–7) (48/48)
CSF WBC count (× 10^6 ^cells/l)	3380 (767–6,528)*^†‡§ ^(31/31)	359 (128–1,066)^‡§ ^(20/20)	139 (61–366)^§ ^(59/59)	57 (13–102)^§ ^(11/11)	1 (1–2) (50/50)
CSF PMNs (× 10^6 ^cells/l)	2,727 (398–5,404)*^†‡§ ^(30/31)	305 (112–600)^†‡§ ^(20/20)	25 (7–75)^§ ^(56/59)	5 (2–29)^§ ^(9/11)	< 1
CSF mononuclear (× 10^6 ^cells/l)	248 (38–523)^§ ^30/31	16 (6–93)^§ ^(20/20)	74 (25–327)^§ ^(56/59)	79 (25–106)^§ ^(9/11)	< 1
CSF glucose (mmol/l)	2.2 (0.6–3.5)^†§ ^(30/31)	3.3 (3.0–3.7)^§ ^(17/20)	3.3 (3.0–3.6)^§ ^(56/59)	3.1 (2.7–3.9) (10/11)	3.7 (3.4–4.5) (48/50)
CSF/blood glucose ratio	0.3 (0.1–0.5)*^†§ ^(22/31)	0.6 (0.4–0.7) (15/20)	0.6 (0.5–0.7) (46/59)	0.5 (0.3–0.7) (8/11)	0.6 (0.6–0.8) (30/50)
CSF protein (g/l)	1.9 (1.5–4.8)*^†§ ^(30/31)	0.7 (0.7–1.4)^§ ^(20/20)	0.7 (0.4–1.3)^§ ^(54/59)	1.1 (0.6–1.7)^§ ^(11/11)	0.3 (0.2–0.5) (47/50)
Blood WBC count (× 10^9 ^cells/l)	21.7 (14.1–24.4)^†‡§ ^(31/31)	14.4 (8.3–16.8)^† ^(20/20)	8.8 (7.3–10.9) (59/59)	7.4 (6.9–9.6) (9/11)	9.0 (6.2–12.8) (48/50)
Blood PMNs (× 10^9 ^cells/L)	17.1 (12.5–21.5)*^†‡§ ^(30/31)	10.9 (7.0–15.0)^†§ ^(20/20)	6.8 (5.3–8.5) (58/59)	4.5 (4.1–9.2) (4/11)	6.0 (4.5–9.6) (39/50)
Blood lymphocytes (× 10^9 ^cells/L)	1.1 (0.7–1.5) (30/31)	1.4 (1.1–1.8) (20/20)	1.5 (1.0–1.9) (58/59)	1.7 (0.8–2.1) (4/11)	1.3 (0.8–2.1) (38/50)
Blood monocytes (× 10^9 ^cells/l)	0.7 (0.4–1.0)^† ^(30/31)	0.6 (0.4–0.9) (20/20)	0.4 (0.3–0.6) (58/59)	0.6 (0.3–0.9) (4/11)	0.4 (0.3–0.8) (38/50)
Platelets (× 10^9 ^cells/l)	235 (166–286) (31/31)	286 (215–370) (20/20)	254 (210–310) (56/59)	231 (174–351) (9/11)	230 (168–275) (47/50)
Sodium (mmol/l)	136 (135–138) (30/31)	136 (133–139) (20/20)	138 (136–140) (59/59)	130 (127–140) (8/11)	138 (136–141) (43/50)
Potassium (mmol/l)	3.6 (3.3–3.9)^† ^(29/31)	3.8 (3.6–4.1) (19/20)	3.9 (3.7–4.2) (59/59)	4.0 (3.9–4.1) (7/11)	3.9 (3.4–4.3) (44/50)
Creatinine (μmol/l)	76 (54–85) (30/31)	58 (46/72) (18/20)	75 (55–85) (56/59)	83 (73–100) (7/11)	71 (49–87) (45/50)
Factors II, IIV and X	0.57 (0.4–0.7)^† ^(28/31)	0.92 (0.7–1.0) (11/20)	0.78 (0.7–1.0) (36/59)	0.73 (0.6–1.0) (7/11)	0.74 (0.5–0.9) (31/50)
Arterial PO_2 _(kPa)	11.0 (9.0–13.0) (15/31)	12.3 (10.7–13.8) (7/20)	8.5 (7.8–11.5) (3/59)	5.2 (1/11)	12.7 (8.0–15.0) (6/50)
Positive blood culture	62%*^†‡§ ^(18/29)	0% (0/20)	0% (0/59)	0% (0/10)	6% (3/48)

Values are expressed as percentage or median (interquartile range) (*n/n *evaluated). For continuous data Kruskal Wallis test with subsequent Mann-Whitney test was used; for categorical data Fisher's exact test was employed. When appropriate, correction for multiple comparisons with a Bonferroni coefficient of 10 was applied. *P < 0.05 versus purulent meningitis of unknown aetiology; ^†^*P *< 0.05 versus lymphocytic meningitis; ^‡^*P *< 0.05 versus encephalitis; ^§^*P *< 0.05 versus non-meningitis. ^a^For example, malignancy, diabetes, drug abuse or alcoholism. CSF, cerebrospinal fluid; PMN, polymorphonuclear leucocyte; PO_2_, partial oxygen tension; WBC, white blood cell.

### Levels of macrophage migration inhibitory factor in cerebrospinal fluid at admission

**Figure 1:**
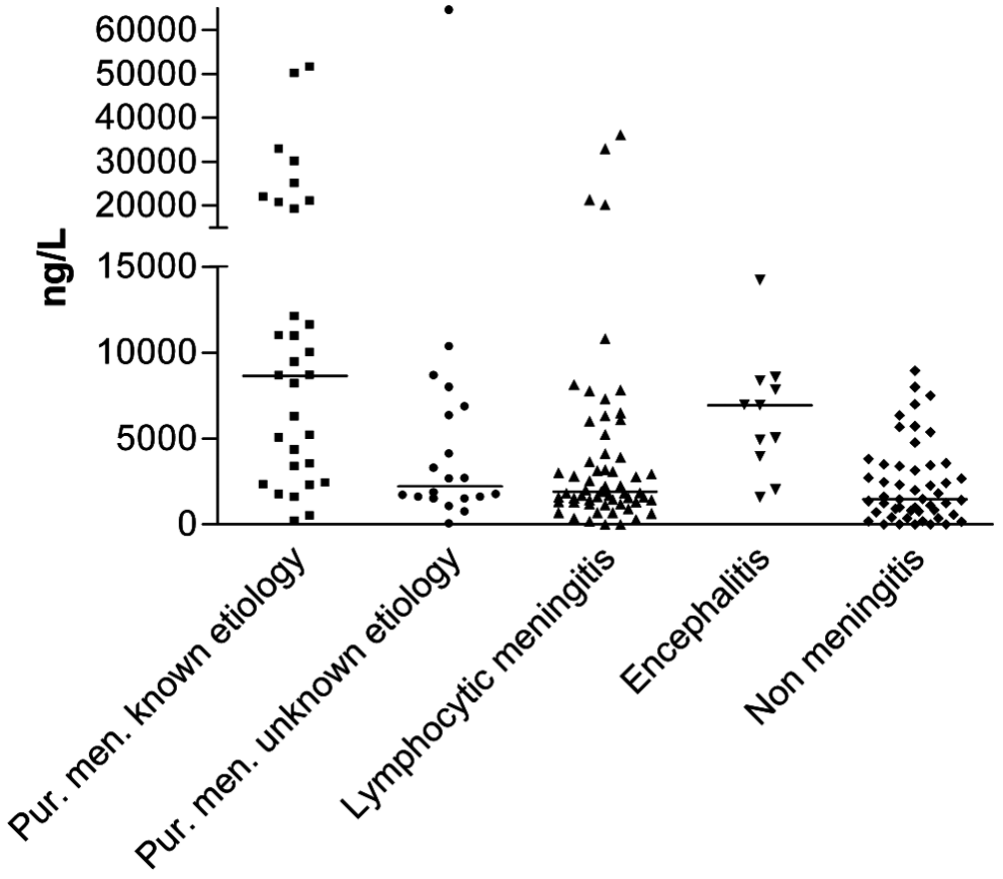
CSF MIF levels from patients with suspected or confirmed meningitis at admission. There was a significant difference between the five diagnostic groups (Kruskal Wallis: *P *< 0.0001). Bars indicate medians. Group 1 includes patients with purulent meningitis of known aetiology (n = 31), group 2 purulent meningitis of unknown aetiology (n = 20), group 3 lymphocytic meningitis (n = 59), group 4 encephalitis (n = 11) and group 5 no CNS infection (n = 50). *P *values calculated using the Mann Whitney test. Group 1 versus group 2 (*P *= 0.003) versus group 3 (*P *< 0.001) versus group 4 (*P *= 0.2) versus group 5 (*P *< 0.001). Group 2 versus group 3 (*P *= 0.5) versus group 4 (*P *= 0.04) versus group 5 (*P *= 0.055). Group 3 versus group 4 (*P *= 0.004) versus group 5 (*P *= 0.075). Group 4 versus group 5 (*P *< 0.001). CSF, cerebrospinal fluid; MIF, macrophage migration inhibitory factor.

A total of 163 patients had measurable CSF MIF levels, whereas eight patients had MIF levels under 20 ng/l. There was a significant difference between the five diagnostic patient groups (Kruskal Wallis test, *P *< 0.0001; Figure 1). Significantly higher CSF MIF levels were detected in patients with purulent meningitis of known aetiology (8,639 [3,344 to 20,600] ng/l) than in those with purulent meningitis of unknown aetiology (2,209 [1,516 to 6,550] ng/l; Mann Whitney test, *P *= 0.003), those with lymphocytic meningitis (1,912 [1,302 to 4,105] ng/l; *P *< 0.001) and those suspected of having meningitis but without evidence of CNS infection (1,472 [672 to 3,447] ng/l; *P *< 0.001). Also, patients with encephalitis (6,937 [3,961 to 8,353]) had significantly higher CSF MIF levels than did those with lymphocytic meningitis (*P *= 0.004) and patients without meningitis (*P *< 0.001). Moreover, patients with pneumococcal meningitis had significantly higher CSF MIF levels than did those with meningococcal meningitis (11,569 ng/l [8,615 to 21,935] ng/l versus 5,006 [1,717 to 10,905] ng/l; *P *= 0.02). Before lumbar puncture, two out of 171 patients – one with pneumococcal meningitis (CSF MIF level 51,539 ng/l) and one with VZV encephalitis (CSF MIF level 5,042 ng/l) received therapy with prednisolone 5 mg/day for treatment of rheumatoid arthritis and chronic myeloid leukaemia, respectively.

### Relation between MIF levels in CSF and blood

One patient with VZV encephalitis had approximately fivefold higher MIF levels in CSF than in blood (5,042 ng/l versus 1,031 ng/ml), and one patient with pneumococcal meningitis with accompanying bacteraemia had lower MIF levels in CSF than in blood (8,615 ng/l versus 10,913 ng/l). Three patients without CNS infection had lower MIF levels in CSF than in blood (one patient with pneumonia [2,660 ng/l versus 4,955 ng/l, respectively] and two with fever of unknown origin [366 ng/l versus 1,371 ng/l and 83 ng/l versus 1,864 ng/l]), whereas one patients with hepatitis due to cytomegalovirus had higher MIF levels in CSF than in blood (1,989 ng/l versus 207 ng/l).

### Associations of CSF MIF levels with clinical features

#### Purulent meningitis

Among patients with purulent meningitis, CSF MIF levels were to some degree associated with severity of the infection. CSF MIF levels were significantly higher in patients who required assisted ventilation than in those who did not (10,493 [5,961 to 22,725] ng/l versus 3,240 [1,563 to 9,302] ng/l; *P *= 0.003), and in patients with impaired consciousness than in those without (8,614 [3,344 to 20,935] ng/l versus 2,625 [1,561 to 7,530] ng/l; *P *= 0.02). However, CSF MIF levels were not significantly higher in patients who died from the infection than in surviving patients (6,662 [3,383 to 17,855] ng/l versus 5,006 [1,701 to 10,971] ng/l, respectively; *P *> 0.05) or in patients who developed septic shock than in those who did not (5,161 [3,830 to 25,474] ng/l versus 5,617 [1,693 to 10,922] ng/l; *P *> 0.05). In purulent meningitis of known bacterial aetiology, the following were all associated with high CSF MIF levels: positive Gram stain (10,905 [6,227 to 20,935] ng/l versus 2,821 [772 to 4,111] ng/l for negative Gram stain; *P *= 0.006), positive CSF culture (10,905 [5,161 to 20,935] ng/l versus 2,608 [733 to 5,750] ng/l for negative CSF culture; *P *= 0.008) and positive blood culture (11,270 [8,502 to 21,185] versus 4,315 [2,251 to 6,227] ng/l for negative blood culture; *P *= 0.006).

#### Encephalitis

The two patients who died from encephalitis had high CSF MIF levels (6,937 ng/l and 14,213 ng/l), but the difference in CSF MIF between these patients and those who survived (5,042 [2,999 to 8,090] ng/l) was not statistically significant (*P *> 0.05).

#### Patients suspected of meningitis but without evidence of CNS infection

CSF MIF levels correlated with number of days hospitalized (rho = 0.31, *P *= 0.03).

### Association of CSF MIF levels with biochemical parameters in CSF and blood

Associations between CSF MIF levels at admission and biochemical parameters in CSF and blood are summarized in Table 2. In patients with purulent meningitis of known aetiology, in patients with lymphocytic meningitis and in patients with encephalitis, CSF MIF levels correlated significantly with meningeal inflammation (*P *< 0.05) but not with the systemic inflammatory response (*P *> 0.05). In contrast, no such association was observed in patients with purulent meningitis of unknown aetiology (*P *> 0.05). In patients without CNS infection, CSF MIF correlated significantly with CSF protein levels (*P *< 0.05).

**Table 2:** Association (rho values) between CSF MIF levels at admission and biochemical parameters in CSF and blood

CSF samples	Purulent meningitis of known aetiology (n = 31)	Purulent meningitis of unknown aetiology (n = 20)	Lymphocytic meningitis (n = 59)	Encephalitis (n = 11)	No CNS infection (n = 50)
CSF WBC count	0.49^†^	-0.21	0.35^†^	0.61*	0.07
CSF PMNs	0.50^†^	-0.20	-0.04	0.88^†^	-0.24
CSF mononuclear	0.39*	-0.44	0.37^†^	0.18	0.13
CSF glucose	-0.55^‡^	0.17	-0.04	-0.25	0.14
CSF/blood glucose	-0.51*	-0.22	0.12	-0.21	-0.16
CSF protein	0.42*	0.12	0.31*	0.36	0.39^†^
CSF lactoferrin	0.45*	-0.33	0.15	0.95*	0.20
CSF neopterin	0.32	-0.14	0.33*	0.80	0.17
Blood WBC count	0.13	-0.19	-0.18	-0.50	0.06
Blood neutrophils	0.22	-0.14	-0.18	-0.40	-0.04
Blood lymphocytes	-0.27	-0.29	0.04	-0.40	0.02
Blood monocytes	0.18	-0.60*	-0.14	-0.40	0.11

*P < 0.05, ^†^*P *< 0.01, ^‡^*P *< 0.001. CNS, central nervous system; CSF, cerebrospinal fluid; MIF, macrophage migration inhibitory factor; PMN, polymorphonuclear leucocyte; WBC, white blood cell.

## Discussion

In the present study we found that CSF MIF levels were significantly higher in patients with purulent meningitis and encephalitis than in patients with lymphocytic meningitis and patients suspected of having meningitis but without evidence of CNS infection. In accordance with our findings, a previous study [13] showed that patients with encephalitis due to West Nile virus had elevated CSF MIF levels, compared with uninfected control patients. However, because of significant overlap in CSF MIF levels between the five diagnostic groups identified in the present study, resulting in poor prognostic sensitivity and specificity (data not shown), the use of CSF MIF levels for diagnostic purposes cannot be recommended. In particular, CSF MIF levels were not useful in differentiating between purulent meningitis of unknown aetiology and lymphocytic meningitis.

CSF MIF levels were significantly higher in patients with pneumococcal meningitis than in those with meningococcal meningitis. Pneumococcal meningitis carries a higher mortality than meningococcal meningitis, and differences in bacterial virulence factors may account for higher CSF MIF levels in pneumococcal meningitis than in meningococcal meningitis. Moreover, CSF MIF levels were to some degree related to disease severity, being significantly higher levels in patients with purulent meningitis who were unconscious or who required assisted ventilation, but CSF MIF levels were not correlated with mortality or presence of septic shock. Previous studies found that serum levels of MIF were related to mortality in sepsis [3-5]. Unfortunately, we did not measure serum levels of MIF in the present study, so we were unable to clarify whether serum levels are a better prognostic marker than CSF levels.

In the present study, we found a significant correlation between CSF MIF levels and CSF WBC count in meningitis and encephalitis patients, whereas no association was found between CSF MIF levels and the systemic inflammatory response (blood WBC count). Moreover, Arjona and coworkers [13] found that MIF levels were approximately 10-fold higher in CSF than in plasma in patients with encephalitis due to West Nile virus, indicating that MIF may be locally released during CNS infection. Here we found a fivefold higher MIF concentration in CSF than in blood in the only encephalitis patient in whom we obtained corresponding CSF and blood samples. Also corroborating the findings of previous studies [13], in a limited number of patients with no CNS infection we found that CSF MIF levels were lower than corresponding blood MIF levels. In one patient with pneumococcal meningitis and bacteraemia with a lung focus, high MIF levels were found both in CSF and blood, suggesting that MIF may be elevated at several sites of an infection. However, further studies should be performed to clarify the release of MIF in CSF and blood during meningitis.

MIF is produced by a wide range of cell types including macrophages [18] and activated T cells [19]. Interestingly, encephalitis patients had high CSF MIF levels despite a minor CSF cellular infiltrate, as compared with meningitis patients, which could indicate that MIF was derived from resident brain cells rather than from CSF inflammatory cells. However, the strongest correlation with MIF in encephalitis was found to be for CSF polymorphonuclear leucocytes and CSF lactoferrin (a matrix protein of polymorphonuclear leukocyte-specific granules [20]), whereas the correlation with neopterin (a marker of CNS macrophage activation [21]) was weaker. Indeed, in a study of cerebral malaria conducted in Malawi [22], immunohistochemical analysis of a few fatal bacterial meningitis cases revealed that MIF was primarily expressed in the inflamed meninges, to some degree in astrocytes and ependymal cells, and less frequently in blood vessels with in the brain parenchyma.

Apart from its critical role in sepsis through potentiating septic shock, MIF is a pituitary-derived antagonist of glucocorticoids [6]. Adjunctive therapy with corticosteroids might have a beneficial effect in patients with purulent meningitis who have high CSF MIF levels. However, we found no such association in the present study (data not shown). On the other hand, administration of corticosteroids to rats was found to increase MIF expression in blood [23] as well as various body tissues [24]. Only two patients were treated with glucocorticoids at the time of lumbar puncture; thus, although both patients had high CSF MIF levels, further studies are required to determine the effect of glucocorticoids on CSF MIF release during CNS infection.

The role played by MIF has been studied extensively in experimental disease models using pharmacological intervention (stimulation with MIF or inhibition with antibodies to MIF) or by use of MIF gene-deficient animals. Inhibition or lack of MIF attenuated development of septic shock [3,6], encephalitis [13], autoimmune encephalomyelitis [16,17], rheumatoid arthritis [25,26], colitis [27], concanavalin A induced liver injury [28], glomerulonephritis [29] and atherosclerosis [30]. Experimental meningitis studies have documented that both the systemic and meningeal inflammatory response plays a crucial role in the development of brain damage [7,31]. Therefore, to explore further the role played by MIF in bacterial meningitis, experimental meningitis studies with MIF intervention are still warranted.

## Conclusions

MIF levels were significantly increased in CSF of patients with purulent meningitis of known aetiology or with encephalitis, and they were to some degree associated with severity of the infection. Our findings indicate that MIF may play a pathophysiological role in CNS infection.

## Key messages

• CNS infections cause increased CSF levels of MIF.

• Patients with purulent meningitis of known aetiology or with encephalitis had significantly higher CSF MIF levels than did patients with lymphocytic meningitis or patients with no CNS infection.

• CSF MIF levels were associated with disease severity in patients with purulent meningitis.

## Abbreviations

CNS: central nervous system; CSF: cerebrospinal fluid; ELISA: enzyme-linked immunosorbent assay; MIF: macrophage migration inhibitory factor; PBS: phosphate-buffered saline; VZV: varicella zoster virus; WBC: white blood cell.

## Competing interests

The authors declare that they have no competing interests.

## Authors' contributions

CØ designed the study, collected and analyzed data, and drafted the manuscript. TB participated in collection of data and in revising the manuscript.
